# *Leishmania donovani* populations in Eastern Sudan: temporal structuring and a link between human and canine transmission

**DOI:** 10.1186/s13071-014-0496-4

**Published:** 2014-11-20

**Authors:** Rania Baleela, Martin S Llewellyn, Sinead Fitzpatrick, Katrin Kuhls, Gabriele Schönian, Michael A Miles, Isabel L Mauricio

**Affiliations:** Department of Pathogen Molecular Biology, Faculty of Infectious and Tropical Diseases, London School of Hygiene and Tropical Medicine, Keppel Street, WC1E 7HT London, UK; Institut für Mikrobiologie und Hygiene, Charité Universitätsmedizin, Berlin, Germany; Current address: Department of Zoology, Faculty of Science, University of Khartoum, PO Box 321, Khartoum, Sudan; Current address: Molecular Ecology and Fisheries Genetics Laboratory, School of Biological Sciences, University of Wales, Bangor, Deiniol Road, Bangor, Gwynedd LL57 2UW UK; Current address: Division of Molecular Biotechnology and Functional Genetics, Technical University of Applied Sciences Wildau, Hochschulring 1, 15745 Wildau, Germany; Current address: Instituto de Higiene e Medicina Tropical/Unidade de Parasitologia e Microbiologia Médicas, UEI Parasitologia Médica, Rua da Junqueira, 100, 1349-008 Lisbon, Portugal

## Abstract

**Background:**

Visceral leishmaniasis (VL), caused by the members of the *Leishmania donovani* complex, has been responsible for devastating VL epidemics in the Sudan. Multilocus microsatellite and sequence typing studies can provide valuable insights into the molecular epidemiology of leishmaniasis, when applied at local scales. Here we present population genetic data for a large panel of strains and clones collected in endemic Sudan between 1993 and 2001.

**Methods:**

Genetic diversity was evaluated at fourteen microsatellite markers and eleven nuclear sequence loci across 124 strains and clones.

**Results:**

Microsatellite data defined six genetic subpopulations with which the nuclear sequence data were broadly congruent. Pairwise estimates of F_ST_ (microsatellite) and K_ST_ (sequence) indicated small but significant shifts among the allelic repertoires of circulating strains year on year. Furthermore, we noted the co-occurrence of human and canine *L. donovani* strains in three of the six clusters defined. Finally, we identified widespread deficit in heterozygosity in all four years tested but strong deviation from inter-locus linkage equilibrium in two years.

**Conclusions:**

Significant genetic diversity is present among *L. donovani* in Sudan, and minor population structuring between years is characteristic of entrenched, endemic disease transmission. Seasonality in vector abundance and transmission may, to an extent, explain the shallow temporal clines in allelic frequency that we observed. Genetically similar canine and human strains highlight the role of dogs as important local reservoirs of visceral leishmaniasis.

**Electronic supplementary material:**

The online version of this article (doi:10.1186/s13071-014-0496-4) contains supplementary material, which is available to authorized users.

## Background

Visceral leishmaniasis (VL) is caused by parasites of the *Leishmania donovani* complex. The *L. donovani* complex is distributed throughout Asia, North Africa, Latin America and Southern Europe, affecting mostly vulnerable and neglected populations. Infection is spread via the bite of haematophagous phlebotomine sand fly species, while the role of non-human reservoir hosts varies from region to region [1,2]. The most important endemic foci in terms of prevalence, morbidity and mortality are located in India, Sudan and Brazil. Leishmaniasis is likely to have been endemic to Sudan since antiquity (e.g. Zink et al. [3]). Epidemic outbreaks are periodically reported (e.g. Dereure et al. [4]) with high mortality (e.g. Seaman et al. [5]). Recent surveys of disease burden still show consistently high infection and mortality rates in Eastern Sudan, with up to 16% of all deaths attributed to VL regionally [6]. Infection rates in Sudan are thought to be seasonal, linked to moisture and sand fly abundance [7].

Molecular studies, such as the analysis of the ribosomal DNA internal transcribed spacer (ITS) [8,9], multilocus sequence typing (MLST) [10,11] and multilocus microsatellite typing (MLMT) [12], have shown that VL in Sudan, and the contiguous focus in Ethiopia, is caused by one to two genetic groups of *L. donovani*, distinct from *L. infantum* and other *L. donovani* genetic groups. Nevertheless, unlike on the Indian subcontinent, where an emergent epidemic clone seems responsible for most cases, there is significant genetic diversity within Sudanese *L. donovani* [12-14]. More recently, MLMT typing of Sudanese *L. donovani* has focussed on the role genetic recombination might have in influencing local patterns of population genetic diversity [15]. Genetic recombination in the field and laboratory is increasingly reported within and between *Leishmania* species, with important consequences in terms of vector compatibility and the spread of drug resistance [16-19]. Several studies based on MLMT have used widespread homozygosity within populations as a proxy for inbreeding in *Leishmania*, in the face of widespread linkage disequilibrium and irrespective of whether parasites undergo ‘classic’ (Mendelian) gametic sex [15,20,21],

In the current study we evaluated the genetic diversity of *L. donovani* in Sudan using MLST and MLMT markers in parallel, with special focus on longitudinal patterns of parasite genetic diversity in the hyperendemic village Barbar El Fugara of the Atbara River Region and around it, 1993–2001. We successfully incriminated dogs as important reservoirs of *L. donovani* locally, by comparisons to local strains isolated from patients. Furthermore, we were able to show significant, but minor, subdivision between *L. donovani* isolated from different years based on MLMT and MLST, which we discuss in the light of VL epidemiology. We found evidence for excess homozygosity across all populations and associated linkage disequilibrium, but based on the available data we are unable to attribute this pattern of diversity to either genetic exchange (inbreeding) or gene conversion.

## Methods

### Ethical statement

Sampling in Barbar El Fugara was approved by both the Federal and Gedarif State Ministries of Health and by the Faculty of Medicine, Khartoum University. Informed consent was obtained from the district authorities and from the village committee as well as from all the adults who participated in the study. For younger children the consent was obtained from their parents. Other samples included in this analysis were archival or reference strains.

### Strains, reference strains and clones

A panel of 124 *L. donovani* strains and clones was assembled (Additional file 1: Table S1). Twenty-three strains were biologically cloned (cultures founded from a single organism - one to four clones per strain) on solid media in 3.5 cm Petri dishes incubated at 24°C, using a protocol adapted from Yeo et al. [22]. All but one sample selected for cloning originated from the Atbara River region, and our aim was to facilitate the identification of local hybrids among contemporary circulating strains. As such, cloning was undertaken to eliminate the possibility that heterozygous microsatellite loci or SNPs were the result of mixed infections and clones are indicated in Additional file 1: Table S1. Most strains were collected in the Atbara River region of Eastern Sudan in and around the village of Barbar El Fugara. Sudanese strains were collected from human and canine hosts over an eight-year period (1993–2001). However, further Sudanese samples prior to and after this period were also included for reference. In addition, a geographically representative selection of strains collected from Europe, East Africa and the Middle East was included for comparison. A subset of those strains sampled from Barbar El Fugara has been analysed previously (Additional file 1: Table S1) via MLMT but with different markers to those employed here [15].

### Multilocus microsatellite typing and analysis

**Figure 1:**
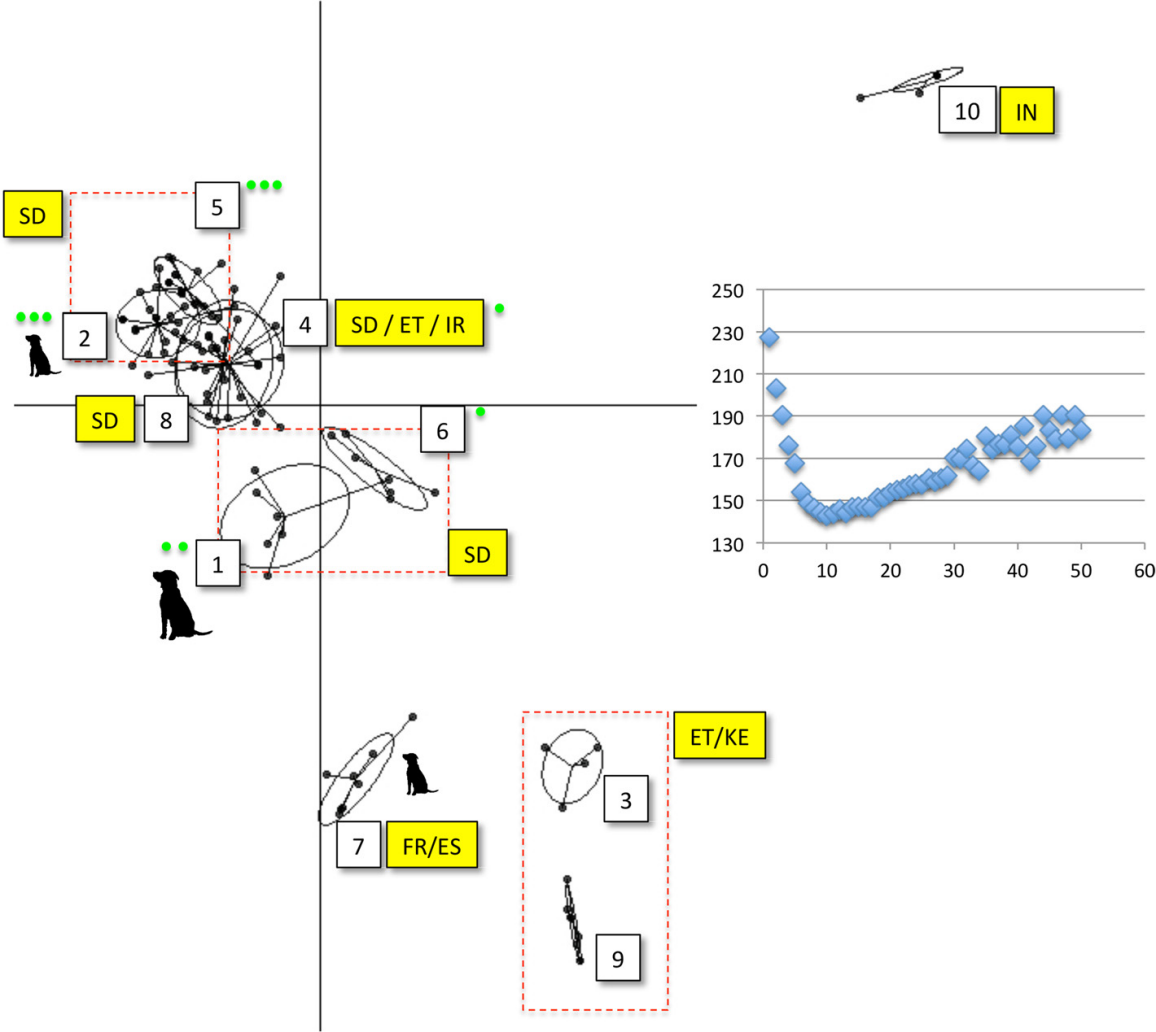
**Genetic clustering among Sudanese and geographically representative** ***L. donovani*** **strains.** The multidimensional scaling plot shows a discriminant analysis of principal components based on 14 microsatellite loci. The optimal number of populations (10) is defined in the Bayesian information criterion (BIC) curve on the right hand side, by the ‘elbow’ of the BIC (y axis) *vs* population number (x axis) curve. Seven principal components were retained, explaining 80% of the total variation. Numeric labels correspond to population identities (see Additional file 1: Table S1 for further details). Yellow boxes indicate country of origin: SD – Sudan, IN-India, KE-Kenya, ET-Ethiopia, PT-Portugal, FR-France, ES-Spain. Red dashed boxes are used to table groups of clusters. Dog symbols alongside populations or dashed boxes indicate the presence of canine *L. donovani* strains. Green circles (1–3 in proportion to abundance) indicate clusters to which strains from Barbara El Fugara belong.

Fragment length analysis of 14 microsatellite markers was undertaken as previously described in Kuhls et al*.*, [12] with the exception of locus CS19 which failed to amplify in our study. Positive controls (HU3 and DD8) and negative controls (i.e. reactions lacking DNA) were included in each set of PCR amplifications and subsequent analyses to ensure compatibility across data sets. To define *a posteriori* the number of putative populations in the data set using a non-parametric (free from Hardy-Weinberg constraints) approach, we employed a *K*-means clustering algorithm, implemented in adegenet [23]. As such, the ‘true’ number of populations can be defined by reference to the Bayesian Information Criterion (BIC), which reaches a minimum when the best-supported assignment of individuals to the appropriate number of clusters is approached. In practice, this number is selected at the ‘elbow’ of the BIC curve (Figure 1). The relationship between these clusters and the individuals within them was evaluated via a discriminant analysis of principal components (DAPC) [24]. We chose to retain the number of principal components (PCs) that represented the first 80% of the total variation in the data set. DAPC results are presented as multidimensional scaling plots in Figure 1. Individual level sample clustering was defined via a neighbour-joining tree based on pairwise distances between multilocus genotypes MLGs [evaluated using *D*_AS_ (1 − proportion of shared alleles at all loci/*n*)] calculated in MICROSAT ([25].

Population-level analyses of microsatellite data were undertaken exclusively on Sudanese *L. donovani* based on populations defined *a priori* by year 1993, 1997, 1998, and 2001 (Additional file 1: Table S1). First we undertook to estimate the level of gene flow between years in Arlequin v3.5 using *F*_*ST*_ (equivalent to Weir and Cockerman’s 1984 estimator (*θ*_*w*_) [26]) and tested this value for significance using a non-parametric random permutation procedure [27]. Secondly, we linearised these values as in Slatkin, 1995, to facilitate direct comparison between values for population pairs [28]. Finally, we calculated population-specific statistics by year: sample size corrected allelic richness (A_r_) in FSTAT 2.9.3.2 [29] and *F*_*IS*_ (an index of the distribution of heterozygosity within and between individuals), per locus per population, also in FSTAT 2.9.3.2. Tests for population-level deviation from Hardy-Weinberg allele frequencies were calculated in Arlequin v3.5 and associated significance levels for p values derived after sequential Bonferroni correction to minimise the likelihood of Type 1 errors. Linkage disequilibrium was defined via the Index of Association and calculated exclusively from biological clones in two populations.

### Multilocus sequence typing and analysis

Direct DNA sequences were generated from the PCR amplification products of eleven MLST targets. Targets included four housekeeping genes previously identified as suitable markers: *asat-Ch24*, *asat-Ch35*, *fh-Ch24, gp63-mspC*) [10,30], and seven new targets developed in the current study. The new targets include a housekeeping gene *cytb5R-Ch22II* (LinJ.22.0590), 4 hypothetical protein-coding genes (*LinJ.01.0010, LinJ.28.0190, LinJ.34.0550* and *LinJ.36.1190*) and 2 pseudogenes (*LinJ.11.0280* and *LinJ.36.0350*). PCR reactions to amplify these targets were undertaken in a final volume of 25 μl, comprising: 2.5 μl 10× buffer, 0.05 mM MgCl_2_, 2.5 μl 0.8 mM dNTPs, 12.5 pmol of each primer, 1.25 U *Taq* polymerase and 25 ng genomic DNA. Amplification conditions were: 30 cycles at 95°C for 1 min, 54–62°C (dependent on primer (Additional file 2: Table S2)) for 1.5 min and 72°C for 1.5 min, with a final extension at 72°C for 10 min. Amplification of targets *LinJ.01.0010*, *LinJ.11.0280*, *LinJ.34.0550*, *LinJ.36.0350* and *LinJ.36.1190* required 10% DMSO. Direct sequencing reactions were performed with internal primers, BigDye^™^ terminator cycle sequencing V3.1 kits (ABI PRISM® Applied Biosystems) and analysed in an ABI PRISM^™^ 3730 DNA sequencer (Applied Biosystems), according to the manufacturer’s instructions. Sequences were inspected and edited visually in Chromas Lite (Copyright © 2005 Technelysium Pty Ltd) and assembled using ClustalW [31] in BioEdit v 5.0.6 [32]. Haplotype phases were reconstructed using the software PHASE v. 2.1.1 [33]. MLST data were analyzed as two separately concatenated target haplotype datasets: coding and hypothetical (10995 bp in total) and non-coding pseudogenes (2173 bp in total). Genome sequences of the reference strains *L. major* MHOM/IL/80/Friedlin and *L. infantum* MCAN/ES/98/LLM-877 were obtained from www.genedb.org. Haplotypes for both coding and non-coding sequence datasets were scanned for mosaic breakpoints in RDP [34]. Sequences for each gene were submitted to Genbank (Accession numbers: FR775540.1-FR775754.1, FR796277.1-FR846362.2, HE648217.1-HE648270.1).

**Figure 2:**
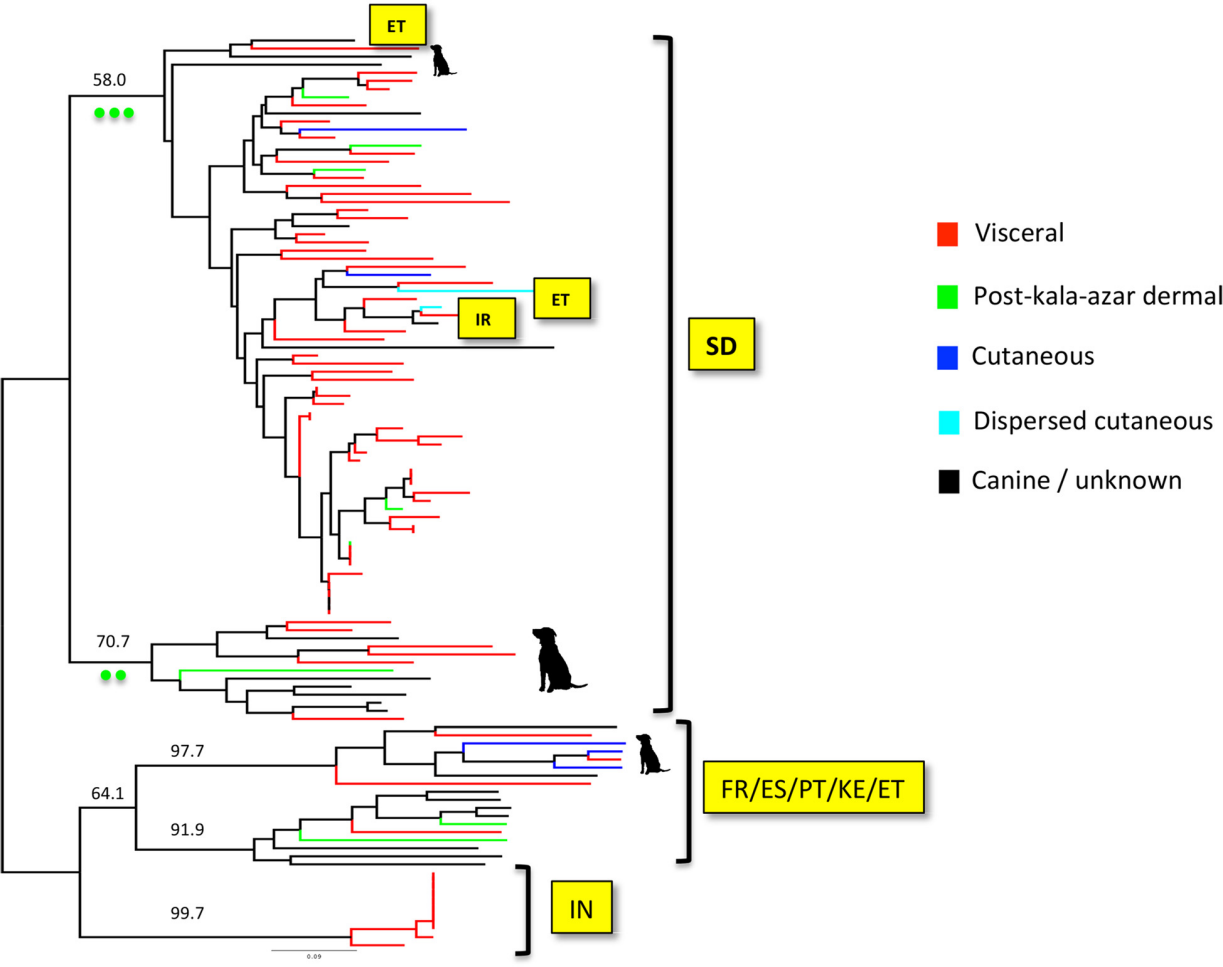
**Maximum likelihood (ML) tree based on phased and concatenated** ***L. donovani*** **haplotypes across eight coding loci (10.9 kb in total).** The ML substitution model adopted was the General Time Reversible plus Gamma with Invariable sites. ML bootstrap support is given in bold. Non-bold bootstraps are derived from the distance based (F84 + Gamma model) neighbor-joining tree from the same dataset. Non-Sudanese strains are identified by country code: SD – Sudan, IN-India, KE-Kenya, ET-Ethiopia, PT-Portugal, FR-France, ES-Spain. Sudanese strains from 1997 (blue), 1998 (yellow), 1999 (orange) and 2001 (red) are labelled. Population codes on the left hand site correspond to those identified via microsatellites (Figure 1).

**Figure 3:**
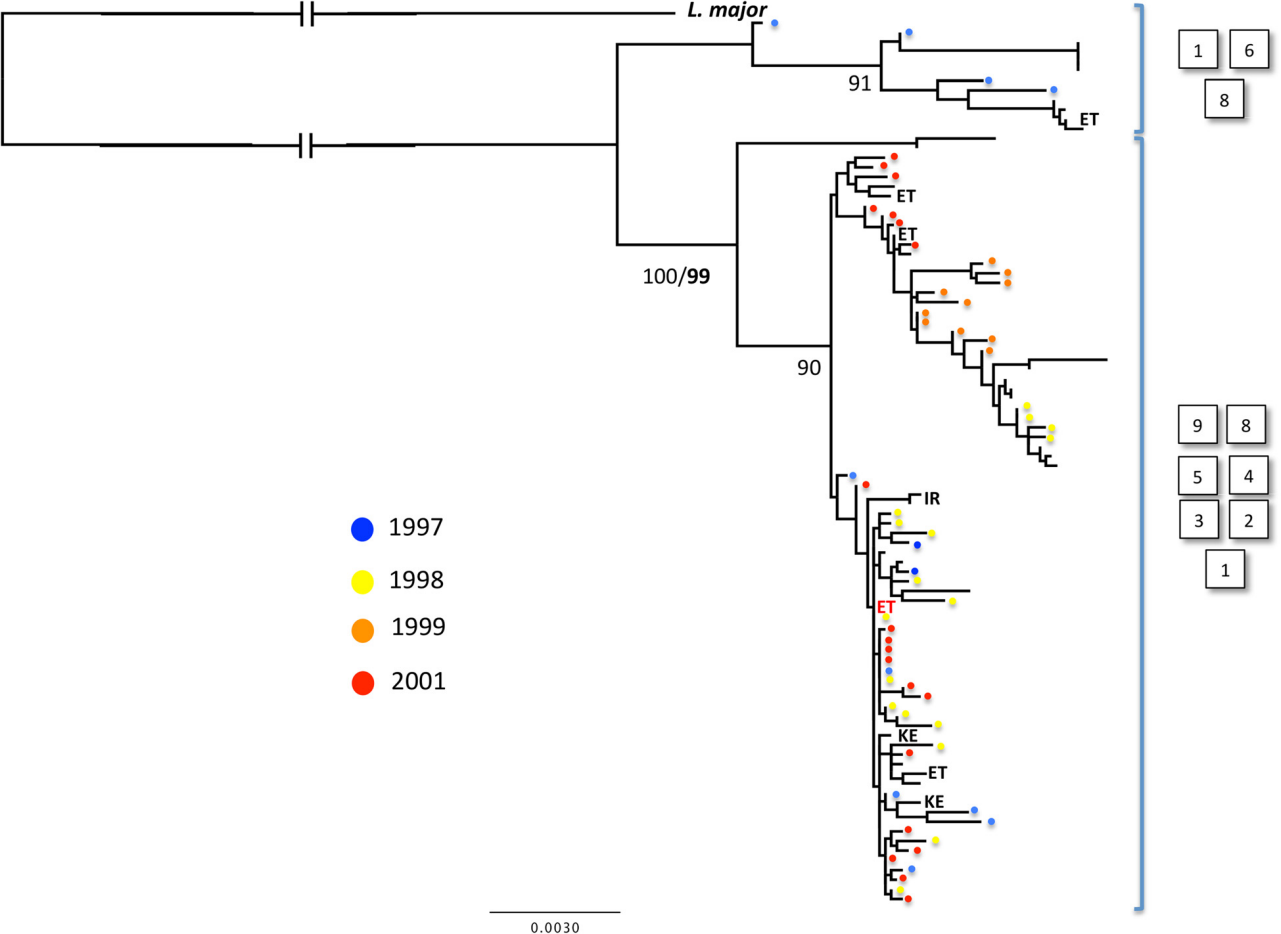
**Unrooted maximum likelihood (ML) tree based on Sudanese** ***L. donovani*** **diplotypes across ten loci (13 kb in total).** Only samples with corresponding microsatellite profiles were included. Eight coding and two pseudo-genes were analysed. ML bootstraps are given in bold, those not in bold are from a distance based (F84 + Gamma model) neighbor-joining analysis of the same dataset. Canine strains are indicated. Sudanese strains from 1993 (dark blue), 1997 (pale blue) 1998 (yellow) and 2001 (red) are labelled.

Phylogenetic and population genetic analysis of various sequence and population sets were undertaken. Phylogenies were inferred using Maximum-Likelihood (ML) implemented in PhyML (4 substitution rate categories) [35]. The best-fit model of nucleotide substitution was selected from 88 models and its significance evaluated according to the Akaike Information Criterion (AIC) in MODELTEST 1.0 [36]. Bootstrap support for clade topologies was estimated following the generation of 100 pseudo-replicate datasets. Neighbour-joining phylogenies were also inferred from the same alignments, and bootstrap support over 1000 pseudo-replicates on congruent clades added to the ML phylogeny. The resultant trees are shown in Figures 2 and 3.

Population genetic differentiation between strains from years 1997, 1998, 1999 and 2001 was calculated from sequence haplotypes in DNAsp [37] using *K*_ST_. *K*_ST_ compares the expected number of nucleotide differences between a pair of sequences within one population with a pair taken across all populations [38]. Statistical significance for observed differentiation was inferred via 10,000 random permutations.

## Results

**Figure 4:**
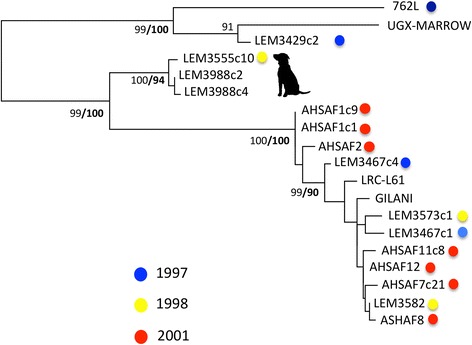
**Microsatellite-based neighbor-joining tree reveals genetic diversity among** ***L. donovani*** **from Sudan.** *D*_AS_ (1-proportion of shared alleles) distances were calculated. Branch colour indicates disease status: red – visceral leishmaniasis; green – post-kala-azar dermal leishmaniasis; blue – cutaneous leishmaniasis, pale blue – dispersed cutaneous leishmaniasis. Dog symbols indicate the presence of strains from canids, green circles indicate the presence of strains from Barbar El Fugara, and country codes are indicated in the yellow boxes: SD – Sudan, IN-India, KE-Kenya, ET-Ethiopia, PT-Portugal, FR-France, ES-Spain. Values indicate % bootstrap support across 1000 pseudo-replicates.

Microsatellite and DNA sequence data were generated across 124 strains and clones. Twenty-two strains were genotyped using both marker classes. Ninety-two were typed using microsatellite markers only, ten with MLST markers only. K-means clustering of the 112 microsatellite profiles revealed ten populations (Figure 1). The majority of Sudanese strains fell into six related populations (2,5,8,4,1,6), while strains from Europe (7) and the Indian subcontinent (10) were clear outliers. A subset of strains from Ethiopia and Iran shared some genetic affinity with those from Sudan. Some other strains from East Africa (ET/KE - 3) were, by contrast, highly divergent. Importantly, canine *L. donovani* from Barbar El Fugara, Sudan, were found alongside strains from humans in four populations (1,2.5,6) across several different sampling years (1997, 1998, 1999, 2000, Additional file 1: Table S1). Clustering observed in Figure 1 was supported by the topology of the neighbour joining tree in Figure 4. Furthermore, no clustering of distinct disease outcome (cutaneous, visceral, post-kala-azar dermal or diffuse cutaneous leishmaniasis, respectively, CL, VL, PKDL or DCL) was observed (Figure 4).

For population level analyses of the microsatellite dataset we focused on only four well represented years from which samples were available: 1993, 1997, 1998 & 2001. Samples from the latter three time points were isolated uniquely from the village of Barbar El Fugara in the hyper-endemic Atbara River region of Eastern Sudan. Estimates for allelic richness over this time period show few radical differences between populations year-on-year (Table 1). Similarly, values for the inbreeding co-efficient *F*_*IS*_ were consistently positive over all loci over the years (Table 1). The index of association, a measure of linkage disequilibrium, was calculated only for years for which ≥8 biological clones were available (1997 and 2001). In both cases, the null hypothesis of random inter-locus associations was strongly rejected (Table 1, *P* <0.0001).

**Table 1:** **Year on year population genetic statistics for Sudanese** ***L. donovani*** **populations**

**Year**	**N**	**Ar**	***F***_***IS***_	**%LociHd**	**%LociHe**	**Ia**
1993	11	2.643 ± 0.289	0.201 ± 0.150	8.3	8.3	ND
1997	11	3.071 ± 0.355	0.596 ± 0.073	46.1	0	ND
1998	14	3.144 ± 0.339	0.479 ± 0.107	46.1	0	2.95 < 0.001
2001	27	2.430 ± 0.291	0.275 ± 0.172	0.33	0.25	2.03 < 0.001

Index of association only calculated for clones.

As well as calculating population diversity statistics within each time point, we were interested in examining the extent of parasite population differentiation longitudinally. We therefore calculated values for pairwise population *F*_*ST*_ and tested for associated significance using a permutation test. Significant structure was detected between all population pairs except years 1997 and 1998 (Table 2). Interestingly, once values for *F*_*ST*_ had been linearised using Slatkin’s correction, the extent of subdivision between time points increased in proportion to the time elapsed between sample collections dates (Table 3).

**Table 2:** **Pairwise** ***F***_***ST***_ **between years suggests incremental shifts in allelic frequencies in Sudanese populations** ***L. donovani***

	**1993**	**1997**	**1998**	**2001**
1993		0.000*	0.000*	0.000*
1997	**0.152**		*0.563*	0.003*
1998	**0.213**	0.008		0.003*
2001	**0.297**	**0.097**	**0.074**	

In bold: statistically significant values of pairwise *F*_*ST*_. In italics, upper left triangle: p values. *represents statistically significant p values.

**Table 3:** **Pairwise** ***F***_***ST***_ **linearised with Slatkin correction**

	**1993**	**1997**	**1998**
1993			
1997	**0.17997**		
1998	**0.27118**	0.00844	
2001	**0.42205**	**0.1072**	**0.07969**

In bold: statistically significant values of pairwise *F*_*ST*_.

In parallel to our analysis of microsatellite fragment size data, we also undertook analysis of DNA sequence data derived from a representative group of 34 strains and clones (including genomic reference strains). Both coding and non-coding regions were scanned for evidence of mosaic breakpoints that might be associated with homologous recombination, which would also potentially disrupt phylogenetic signal in subsequent trees, but no evidence for such events was uncovered. The ML topology derived from coding loci revealed substantial genetic diversity, but little bootstrap support, as one might expect between samples from the same species across a restricted area (Sudan/East Africa, Figure 2). The only clearly divergent clade contained samples classified as population 6, 8 and 1 based on microsatellite typing, as well as a single strain from Ethiopia. Correspondence between sample year and tree topology was limited (Figure 2). As a second approach we examined only those strains for which we had microsatellite data. To improve resolution, we concatenated both coding and non-coding genes and constructed an ML tree from unphased sequence haplotypes (Figure 4). In this case there was a closer match between microsatellite and sequence data. Strains from MLMT-defined populations 6 and 1 were outliers with respect to other Sudanese strains. A notable exception was strain 762 L, which grouped differently between the two sets of markers.

For the sequence data, population genetic analyses were undertaken to explore patterns of diversification across years in samples only from Barbar El Fugara. Among those samples sequenced, the distribution of available data per year was marginally different. Years 1997, 1998, 1999 and 2001 were compared. Pairwise permutations tests suggested significant *K*_ST_ for most population pairs, except 1998/2001, where significance was marginal (*p* =0.0380) (Table 4).

**Table 4:** **Genetic differentiation (** ***K***_**ST**_ **) between years based on concatenated coding sequence data**

	**1997**	**1998**	**1999**
1998	0.115 (0.0090**)		
1999	0.2077 (0.000**)	0.47319 (0.000**)	
2001	0.1079 (0.0050**)	0.03672 (0.0380*)	0.37466 (0.000**)

P-values (in parentheses) are made with reference to 10000 random permutations. Statistical significance: *P < 0.05 and **P < 0.001.

## Discussion

Visceral leishmaniasis in Sudan is a major and on-going public health problem [6]. Molecular epidemiological studies like ours can have a significant role in guiding and informing public health professionals. Our first key observation in this context is that dogs and humans in the region share similar strains. PCR-based and parasitological approaches have already identified dogs as important carriers of *L. donovani* in Sudan [1,4], although circumstantial evidence also points to other truly sylvatic hosts (e.g. [39]). Our high-resolution genetic data clearly demonstrate sharing of parasites between dogs and humans. Previous work on a limited number of the same strains from the same area suggested the possible presence of distinct human and canine transmission cycles [15], however, all three clusters containing canine hosts also contained humans (Figure 1).

The stability of genetic diversity in parasite populations in space is frequently used to infer patterns of regional and global parasite spread (e.g. [40]). Temporal variation in parasite populations can also be highly informative, especially pre- and post- large scale treatment interventions (e.g. [41,42]. The majority of samples we analysed came from an outbreak first reported in 1996 [4]. Given that high rates of infection still occur in the same region today, it is not clear whether ‘outbreak’ successfully described the diseases’ local status [6]. Both our sequence and microsatellite data from different years suggest incremental changes in allelic composition (although, like in an earlier study, no subdivision is detected between years 1997 and 1998 [15]). Mutational instability of highly variable microsatellite markers could play a role. However, it is not clear over what timescale such changes might be expected to happen. In *Trypanosoma cruzi* discrete typing unit I, a related trypanosomatid, two samples taken 20 years apart from the same geographic focus can be identical at 48 microsatellite loci [43]. There are multiple examples in the current dataset where temporally separated strains are closely related to each other. Population 4, for example contains samples from 1967 and 2001. It is, thus, more likely that population processes, such as immigration, founder events and bottlenecks, define the differences between years. However, the shallow clines in allelic composition we observe in the data are not reminiscent of intense serial reductions in parasite population size. Inter-population variation is perhaps more consistent with seasonal changes in infection intensity.

As well as the defining patterns of parasite genetic diversification in the Atbara River region, a secondary goal was to evaluate evidence for genetic exchange among Sudanese *L. donovani* strains. Like previous authors, we were able to detect reduced heterozygosity in the populations studied [15]. However, unlike other authors, we are reluctant to interpret our data as evidence for genetic exchange [15,20,21]. Analysis of cloned *L. donovani* from two populations revealed strong evidence for predominant clonality, despite consistently high values for *F*_*IS*_. Furthermore, sequence data showed no evidence for the mosaics that one might expect to accompany recombination. It is important to state that, although our data do not confirm the presence of genetic exchange, we cannot rule out the occurrence of some recombination/inbreeding, as suggested by Rougeron et al. [15]. We note that extensive genomic-level hybridisation was recently detected among a population of *Leishmania infantum* in Turkey [16], while microsatellite data based on the same strains detected no such phenomenon [44].

## Conclusion

Rapid, low cost, high-resolution genotyping strategies have an important role in elucidating the molecular epidemiology of visceral leishmaniasis, especially where the burden of the disease is felt the most. Population genomic studies of *Leishmania* have now demonstrated the power that such approaches have to reveal the extent and mechanism of genetic exchange in natural populations [16]. It has become apparent that genetic exchange is not a rare event but a feature of natural populations of several *Leishmania* species [16,20,21]; experimental crosses in sand flies suggest Mendelian segregation [17]. Although proof of genetic exchange was not evident among the Sudanese populations analysed here, excess homozygosity occurred in conjunction with LD, and this was interpreted by others as inbreeding [20]. Furthermore, some foci of human and canine *L. donovani* transmission were coincident with overlapping *L. donovani* genotypes, indicating that dogs may have an important role in sustaining human VL in Sudan, which deserves further investigation.

## Additional files

Additional file 1: Table S1. Panel of clones and strains analysed in this study. Additional file 2: Table S2. Primers and annealing temperature for PCR amplification of the new MLST targets.
