# Supplementary material for: Leishmania donovani populations in Eastern Sudan: temporal structuring and a link between human and canine transmission
Source: Parasit Vectors. 2014 Nov 20;7:496. doi: 10.1186/s13071-014-0496-4 (PMC4255451; doi:10.1186/s13071-014-0496-4)
Supplement: Additional file 1: Table S1. — Panel of clones and strains analysed in this study. [file 13071_2014_496_MOESM1_ESM.doc]

Additional file 1: Table S1 Panel of clones and strains analyzed in this study.

| **Country^A^** | **Village^B^** | **Year** | **DAPC** | **Code** | **MLST** | **MLMT** | **Disease^D^** | **Host** |
| --- | --- | --- | --- | --- | --- | --- | --- | --- |
|  |  |  | **Population^C^** |  |  |  |  |  |
| KE | ND | 1954 | 9 | LRC-L53 | NO | YES | ND | Human |
| SD | ND | 1962 | 8 | LRC-L61 | YES | YES | ND | Human |
| SD | ND | 1962 | 8 | 3S | NO | YES | VL | Human |
| KE | ND | 1962 | 9 | LRC-L57 | NO | YES | ND | Insect |
| SD | ND | 1966 | 2 | L46 | NO | YES | VL | Human |
| KE | ND | 1967 | 3 | MRC (L)3 | NO | YES | ND | Human |
| ET | ND | 1967 | 4 | HU3 | NO | YES | VL | Human |
| ET | ND | 1972 | 4 | METTEMA | YES | YES | ND | Human |
| ET | ND | 1972 | 8 | GEBRE1 | NO | YES | VL | Human |
| KE | ND | 1973 | 3 | MRC74 | NO | YES | ND | Human |
| ET | ND | 1975 | 5 | HO174 | NO | YES | ND | Rodent |
| SD | ND | 1975 | 8 | LV139 | NO | YES | CL | Human |
| FR | ND | 1978 | 7 | LEM75 | NO | YES | VL | Human |
| IN | ND | 1980 | 10 | DD8 | NO | YES | VL | Human |
| SD | ND | 1982 | 8 | GILANI | YES | YES | VL | Human |
| KE | ND | 1983 | 9 | NLB189 | NO | YES | PKDL | Human |
| ET | ND | 1984 | 3 | Addis 142 | NO | YES | ND | Human |
| KE | ND | 1984 | 3 | NLB218 | NO | YES | PKDL | Human |
| SD | ND | 1985 | 4 | A22 | NO | YES | VL | Human |
| KE | ND | 1985 | 9 | NLB323 | NO | YES | VL | Human |
| ES | ND | 1986 | 7 | BCN16 | NO | YES | CL | Human |
| ES | ND | 1986 | 7 | LEM935 | NO | YES | CanL | Canine |
| SD | ND | 1987 | 6 | UGX-MARROW | YES | YES | VL | Human |
| FR | ND | 1987 | 7 | RM1 | NO | YES | CanL | Canine |
| CH | ND | 1988 | ND | Turfan 10 | YES | NO | Sand fly | Sand fly |
| ET | ND | 1992 | 4 | CHEMEDAC2^1^ | YES | YES | DCL | Human |
| ET | ND | 1992 | 4 | CHEMEDAC5^1^ | NO | YES | DCL | Human |
| SD | ND | 1992 | 8 | 51-band | NO | YES | VL | Human |
| SD | ND | 1993 | ND | D'Bella | YES | NO | VL | Human |
| SD | ND | 1993 | 8 | 762L | YES | YES | VL | Human |
| SD | ND | 1993 | 6 | GE | NO | YES | VL | Human |
| ES | ND | 1993 | 7 | PM1 | NO | YES | VL | Human |
| SD | ND | 1993 | 8 | 338 | NO | YES | PKDL | Human |
| SD | ND | 1993 | 8 | 35-band | NO | YES | VL | Human |
| SD | ND | 1993 | 8 | 38-UMK | NO | YES | VL | Human |
| SD | ND | 1993 | 8 | 45-UMK | NO | YES | VL | Human |
| SD | ND | 1993 | 8 | 452BM | NO | YES | PKDL | Human |
| SD | ND | 1993 | 8 | 597-2 | NO | YES | PKDL | Human |
| SD | ND | 1993 | 8 | 597LN | NO | YES | PKDL | Human |
| SD | ND | 1993 | 8 | 9S | NO | YES | VL | Human |
| SD | ND | 1993 | 8 | AEB | NO | YES | VL | Human |
| FR | ND | 1995 | 7 | LPN114 | NO | YES | VL | Human |
| IR | ND | 1996 | 4 | MESH-17 | YES | YES | ND | Marsupial |
| IN | ND | 1996 | 10 | THAK35 | NO | YES | VL | Human |
| FR | ND | 1997 | 7 | LSL29 | NO | YES | CL | Human |
| SD | BF | 1997* | 6 | LEM3429 | YES | YES | VL | Human |
| SD | BF | 1997* | 5 | LEM3467 | YES | YES | VL | Human |
| SD | BF | 1997* | 1 | LEM3471 | NO | YES | VL | Human |
| SD | BF | 1997* | 1 | LEM3472 | NO | YES | PKDL | Human |
| SD | BF | 1997* | 2 | LEM3427 | NO | YES | VL | Human |
| SD | BF | 1997* | 2 | LEM3454 | NO | YES | VL | Human |
| SD | BF | 1997* | 2 | LEM3458 | NO | YES | VL | Human |
| SD | BF | 1997* | 2 | LEM3467C15^1^ | NO | YES | VL | Human |
| SD | BF | 1997* | 2 | LEM3473 | NO | YES | PKDL | Human |
| SD | BF | 1997* | 5 | LEM3475 | NO | YES | PKDL | Human |
| SD | BF | 1997* | 6 | LEM3463 | NO | YES | VL | Human |
| ND | BF | 1998 | ND | LEM3575 | YES | NO | VL | Human |
| ND | BF | 1998 | ND | LEM3571 | YES | NO | VL | Human |
| SD | BF | 1998 | 1 | LEM3555 | YES | YES | CanL | Canine |
| SD | BF | 1998* | 2 | LEM3573 | YES | YES | VL | Human |
| SD | BF | 1998* | 5 | LEM3582 | YES | YES | VL | Human |
| SD | BF | 1998* | 1 | LEM3555c6^1^ | NO | YES | CanL | Canine |
| SD | BF | 1998* | 1 | LEM3555c7^1^ | NO | YES | CanL | Canine |
| SD | BF | 1998* | 2 | LEM3556 | NO | YES | CanL | Canine |
| SD | BF | 1998* | 2 | LEM3563C3^1^ | NO | YES | VL | Human |
| SD | BF | 1998* | 2 | LEM3566 | NO | YES | VL | Human |
| SD | BF | 1998* | 2 | LEM3567 | NO | YES | VL | Human |
| SD | BF | 1998* | 2 | LEM3582C3^1^ | NO | YES | VL | Human |
| SD | BF | 1998* | 4 | LEM3556C3^1^ | NO | YES | CanL | Canine |
| SD | BF | 1998* | 5 | LEM3582C1^1^ | NO | YES | VL | Human |
| SD | BF | 1998* | 5 | LEM3582C4^1^ | NO | YES | VL | Human |
| SD | BF | 1998* | 5 | LEM3582C5^1^ | NO | YES | VL | Human |
| SD | BF | 1999 | ND | LEM3787 | YES | NO | CanL | Canine |
| SD | BF | 1999 | ND | LEM3804 | YES | NO | CanL | Canine |
| SD | BF | 1999* | 5 | LEM3785 | NO | YES | CanL | Canine |
| SD | BF | 2000 | ND | LEM3949 | YES | NO | CanL | Canine |
| SD | BF | 2000 | 1 | LEM3948 | NO | YES | CanL | Canine |
| SD | BF | 2000 | 6 | LEM3946 | NO | YES | CanL | Canine |
| PT | ND | 2000 | 7 | IMT260 | NO | YES | CL | Human |
| IN | ND | 2000 | 10 | BHU2 | NO | YES | VL | Human |
| IN | ND | 2000 | 10 | BHU4 | NO | YES | VL | Human |
| IN | ND | 2000 | 10 | BHU5 | NO | YES | VL | Human |
| IN | ND | 2000 | 10 | DEVI | NO | YES | VL | Human |
| SD | BF | 2000* | 1 | LEM3988c1^1^ | YES* | YES | CanL | Canine |
| SD | BF | 2001 | 4 | AHSAF1 | YES | YES | VL | Human |
| SD | BF | 2001 | 2 | AHSAF11 | YES | YES | VL | Human |
| SD | BF | 2001 | 2 | AHSAF12 | YES | YES | VL | Human |
| SD | BF | 2001 | 4 | AHSAF2C6^1^ | YES* | YES | VL | Human |
| SD | BF | 2001 | 2 | AHSAF4 | YES | YES | VL | Human |
| SD | BF | 2001 | 5 | AHSAF7C21^1^ | YES | YES | VL | Human |
| SD | BF | 2001 | 2 | AHSAF8 | YES | YES | VL | Human |
| SD | BF | 2001 | 2 | AHSAF11C10^1^ | NO | YES | VL | Human |
| SD | BF | 2001 | 2 | AHSAF4C8^1^ | NO | YES | VL | Human |
| SD | BF | 2001 | 2 | AHSAF6 | NO | YES | VL | Human |
| SD | BF | 2001 | 4 | AHSAF1 (2T) | NO | YES | VL | Human |
| SD | BF | 2001 | 4 | AHSAF1 (2T) | NO | YES | VL | Human |
| SD | BF | 2001 | 5 | AHSAF13 | NO | YES | VL | Human |
| SD | BF | 2001 | 5 | AHSAF13C12^1^ | NO | YES | VL | Human |
| SD | BF | 2001 | 5 | AHSAF13C2^1^ | NO | YES | VL | Human |
| SD | BF | 2001 | 5 | AHSAF13C9^1^ | NO | YES | VL | Human |
| SD | BF | 2001 | 5 | AHSAF4C5^1^ | NO | YES | VL | Human |
| SD | BF | 2001 | 5 | AHSAF4C9^1^ | NO | YES | VL | Human |
| SD | BF | 2001 | 5 | AHSAF6C10^1^ | NO | YES | VL | Human |
| SD | BF | 2001 | 5 | AHSAF6C11^1^ | NO | YES | VL | Human |
| SD | BF | 2001 | 5 | AHSAF6C12^1^ | NO | YES | VL | Human |
| SD | BF | 2001 | 5 | AHSAF6C7^1^ | NO | YES | VL | Human |
| SD | BF | 2001 | 5 | AHSAF7C22^1^ | NO | YES | VL | Human |
| SD | BF | 2001 | 5 | AHSAF8C10^1^ | NO | YES | VL | Human |
| SD | BF | 2001 | 5 | AHSAF9C2^1^ | NO | YES | VL | Human |
| SD | BF | 2001 | 5 | AHSAF9C5^1^ | NO | YES | VL | Human |
| IN | ND | 2001 | 10 | BHU20140 | NO | YES | VL | Human |
| IN | ND | 2002 | 10 | BHU1 | NO | YES | VL | Human |
| IN | ND | 2002 | 10 | BHU3 | NO | YES | VL | Human |
| IN | ND | 2002 | 10 | BHU6 | NO | YES | VL | Human |
| SD | ND | 2004 | ND | Don134 | YES | NO | VL | Human |
| SD | ND | 2006 | 8 | 1S | NO | YES | VL | Human |
| SD | ND | ND | 4 | KHARTOUM | NO | YES | VL | Human |
| KE | ND | ND | 9 | LRC-L445 | NO | YES | ND | Human |
| ND | ND | ND | ND | LEM221 | YES | NO | ND | ND |
| ND | ND | ND | ND | L. infantum | YES | NO | ND | ND |
| ND | ND | ND | ND | L. major | YES | NO | ND | ND |
| ET | ND | ND | 6 | HUSSEN | YES | YES | VL | Human |
| SD | ND | ND | 2 | SUDAN1 | YES | YES | CL | Human |

^A^SD – Sudan, IN-India, KE-Kenya, ET-Ethiopia, PT-Portugal, FR-France, ES-Spain

^B^BF – Barbara El Fugara

^C^Please refer to the multidimensional scaling plot in Figure 1

^D^VL-Visceral Lieshmaniasis, CanL-Canine Leishmaniasis, PKDL – Post Kalazar Dermal Lesihamiasis, CL- Cutaneous Lesihmaniasis

^E^ MLST profiles were derived from the uncloned strains.

ND – No Data

*Sample previously analyses in Rougeron et al 2011 [21]

^1^Biological clone
