# Supplementary material for: Leishmania donovani populations in Eastern Sudan: temporal structuring and a link between human and canine transmission
Source: Parasit Vectors. 2014 Nov 20;7:496. doi: 10.1186/s13071-014-0496-4 (PMC4255451; doi:10.1186/s13071-014-0496-4)
Supplement: Additional file 2: Table S2. — Primers and annealing temperature for PCR amplification of the new MLST targets. [file 13071_2014_496_MOESM2_ESM.doc]

Additional file 2: Table S2 Primers and annealing temperature for PCR amplification of the new MLST targets

| **Target** | **Forward Primer** | **Reverse Primer** | **Annealing temperature**  **(°C)** |
| --- | --- | --- | --- |
|  | **sequence (5’-3’)** | **sequence (5’-3’)** |  |
| *LinJ.01.0010* | GCTGCTGACAAGATGCGCT | GCAGAGTCGCTTGGTCACT | 56 |
| *LinJ.11.0280* | AGCCGCCGAAACAGCAAG | CCCGTCGCCGAGTCTTTG | 62 |
| *LinJ.28.0190* | GTCGCAGTCCAACTCCCATA | CGCATAGCAAAAGGCAAAA | 56 |
| *LinJ.34.0550* | GTCTCCGCAAACTCTGTTCC | GAACACTCCGCTTCACATGAT | 54 |
| *LinJ.36.0350* | ACTTGGTCTTGGTGGTACGG | TGGAGGACGGAGAGACTTTG | 57 |
| *LinJ.36.1190* | GCTTCTCGCTATTGCTCGTC | ACTGGCAGGCACACATCAG | 56 |
| *cytb5R-Ch22II* | TCGCGCTAATTCTCGTACCA | CTCACAGAGTCAAGGCTT | 60 |
